# Polympact: exploring functional relations among common human genetic variants

**DOI:** 10.1093/nar/gkac024

**Published:** 2022-01-21

**Authors:** Samuel Valentini, Francesco Gandolfi, Mattia Carolo, Davide Dalfovo, Lara Pozza, Alessandro Romanel

**Affiliations:** Department of Cellular, Computational and Integrative Biology (CIBIO), University of Trento, Trento, Italy; Department of Cellular, Computational and Integrative Biology (CIBIO), University of Trento, Trento, Italy; Department of Cellular, Computational and Integrative Biology (CIBIO), University of Trento, Trento, Italy; Department of Cellular, Computational and Integrative Biology (CIBIO), University of Trento, Trento, Italy; Department of Cellular, Computational and Integrative Biology (CIBIO), University of Trento, Trento, Italy; Department of Cellular, Computational and Integrative Biology (CIBIO), University of Trento, Trento, Italy

## Abstract

In the last years, many studies were able to identify associations between common genetic variants and complex diseases. However, the mechanistic biological links explaining these associations are still mostly unknown. Common variants are usually associated with a relatively small effect size, suggesting that interactions among multiple variants might be a major genetic component of complex diseases. Hence, elucidating the presence of functional relations among variants may be fundamental to identify putative variants’ interactions. To this aim, we developed Polympact, a web-based resource that allows to explore functional relations among human common variants by exploiting variants’ functional element landscape, their impact on transcription factor binding motifs, and their effect on transcript levels of protein-coding genes. Polympact characterizes over 18 million common variants and allows to explore putative relations by combining clustering analysis and innovative similarity and interaction network models. The properties of the network models were studied and the utility of Polympact was demonstrated by analysing the rich sets of Breast Cancer and Alzheimer's GWAS variants. We identified relations among multiple variants, suggesting putative interactions. Polympact is freely available at bcglab.cibio.unitn.it/polympact.

## INTRODUCTION

Common genetic variants in the form of Single Nucleotide Polymorphisms (SNPs) and Small Insertions and Deletions (INDELs) are the most frequent forms of DNA polymorphisms. SNPs and INDELs are supposed to be the largest source of phenotypic variation across individuals. Although common variants are mostly located outside of gene coding regions and seem to have no direct consequences on protein sequences and phenotypes, genome-wide association studies (GWAS) identified thousands of them associated with complex traits and diseases (1). Despite expression quantitative trait loci (eQTL) studies have broadly shown that non-coding variants modulate gene expression (2), there are still limited examples of clear mechanistic models linking common variants and biological functions (3,4) and the functional role of most of them remains largely unknown. Indeed, most variants identified in GWAS studies have low effect size, suggesting that individual variants have a small impact on the heritability of complex traits and diseases (5). In addition, complex traits and diseases are often affected by many genes. Overall, this suggest that the interaction among common variants may play an important role and could represents a major genetic component of complex diseases (6).

Advances in high-throughput technologies, especially those based on next-generation sequencing (NGS), have generated a huge amount of genomic datasets of different types. Several databases and web applications have been developed upon these datasets to annotate genetic variants, providing effective platforms for the exploration of their functional properties. Some of these resources are focused on specific aspects of SNPs and INDELs like SNP2TFBS (7), which annotates how variants’ alleles may affect transcription factors (TFs) motifs, or HACER (8), which allows to explore how non-coding variants in active enhancers may modulate gene expression. Other resources instead, like RegulomeDB (9), HaploReg (10) and the recent VARAdb (11), provide extensive annotations of common variants by integrating ChIP-seq data, chromatin accessibility and interaction data, TFs motif changes, eQTLs and GWAS data. Although these resources provide important information to investigate the functional role of single variants, none of them allows to aggregate information of multiple variants, limiting hence their applicability to investigate to what extent different variants may be involved in the modulation of same genes or genes involved in same biological processes. Tools and frameworks to explore functional relations and links among multiple variants are indeed needed and fundamental to help identifying putative variants’ interactions.

To overcome these limitations, we developed Polympact, a computational resource and framework which allows to investigate the presence of functional relations among multiple variants. On the one side, Polympact characterizes over 18 million common, mainly non-coding, variants by combining: (i) cell line and tissues regulatory elements data; (ii) the landscape of changes observed in transcription factors binding sites (TFBS) scores; (iii) the association of genetic variants genotype with the expression of protein coding genes in various healthy human tissues. On the other side, Polympact provides a novel framework to explore functional relations among a set of queried variants, combining clustering analysis, a network model describing similarities which also includes community detection features, and an additional network model which integrates all functional annotations computed and collected in Polympact to explore in detail interactions among variants and genes.

We believe that Polympact could become a useful and effective computational platform to investigate the potential impact of multiple common genetic variants in human diseases and biological processes.

## MATERIALS AND METHODS

### Collection of genetic variants

We collected genetic variants information from dbSNP version 151 (12) using version hg19 as human reference genome. We kept all common variants with Minor Allele Frequency (MAF) greater or equal than one percent, considering the general population frequencies available from 1000 Genomes Project (13) or the TOPMed (14) data. Overall, we collected 18 683 752 genetic variants composed by 14 810 175 SNPs and 3 873 577 INDELs.

### Functional annotation of genetic variants

ChIP-seq data from ENCODE (15) and RoadMap (16) projects (as available in March 2018) were retrieved. We collected data for 9074 narrow peak experiments and 1395 broad peak experiments on 42 tissues and 238 cell lines, annotating 755 functional elements divided between 724 transcription factors and 31 histone marks. Then, using the BEDTools intersect module (17) with default parameters, we checked, for each collected variant, if its genomic position fell within a functional peak in all replicates of a given TF/histone mark specific experiment. Overall, we annotated all the variants by the number of experiments that supports a TF or histone mark in a cell line/tissue. We also annotated variants based on functional marker data available from CONREL (18), a resource we recently developed which provides an extensive collection of consensus promoters, enhancers and active enhancers across 38 tissue types.

### Impact of genetic variants on binding motifs

We retrieved 5424 TFBS consensus motifs in the form of position frequency matrices (PFM) from Transfac Professional (19), Hocomoco (20), Homer (21) and Jaspar (22) and 552 human RNA binding protein (RBP) consensus motifs from RBPDB database (23). Extending an approach we previously proposed and used in (24), for each variant we performed an extensive motif search using a pattern matching approach, considering a 30 bp flanking window around the variant and using the TESS computational tool (25). RBP motifs were used to characterize only UTR variants.

Among the log-likelihood-ratio-based scores provided by TESS we used the score *La*, which represents the log-odds ratio of the match, and the score *Lm*, which represents the maximum possible log-odds ratio. TFBS and RBP scores (hereafter referred to as *binding motifs scores*) were computed considering both the reference genome sequence and the sequence modified with the variant alternative allele. For each motif, significance of scores was determined comparing the calculated scores against a motif-specific reference distribution of scores computed across random genomic sequences. For motifs shorter than 11 nucleotides we enumerated all the possible nucleotide combinations, while for longer motifs we extracted 1 000 000 random sequences from the hg19 human reference genome. Considering all positive scores obtained across all motifs tested at the specific genetic variants, score ratios *La/Lm* were calculated and normalized considering the average of *La/Lm* ratios and the average of length-specific *La/Lm* ratios.

Overall, motif matches at the specific genetic variant locus were retained when: (i) the match overlaps the genetic variant; (ii) the score for the reference allele or the score for the alternative allele was at least six (TESS default parameter) for TFBSs and two for RBPs (which motifs are generally smaller); (iii) the score *P*-value for the reference or the alternative allele calculated against the motif-specific reference distribution is smaller than 0.001; (iv) the normalized *La/Lm* score ratio for the reference or alternative allele is >0.5.

Retained variants were classified as a ‘match’, when the difference between the score computed on the reference sequence and the alternative sequence was <10%, and as a ‘change’, when this difference was equal or >10%. Instead, we call an ‘addition’ when the alternative allele score is positive (and respects all the other thresholds) and the reference allele score is negative, while a ‘deletion’ is called in the opposite case. When the analysed genetic variant is a small insertion and the motif match starts inside the added genomic sequence, we call it an ‘addition’ in all cases. Examples of considered cases are provided in Supplementary Figure S1.

### Integration of TCGA and GTEx projects data

Genotype and transcriptomic information from either TCGA (26) and GTEx (27) datasets were collected and examined. We conducted the analysis across 15 different human tissues for which genotype-expression normal matching samples were provided, including breast, brain, uterus, lung, liver, cervix, prostate, pancreas, stomach, esophagus, thyroid, skin, ovary, colon and bladder. Specifically, genotype and normal RNA-seq samples from each tissue were processed and analysed separately according to the tissue-specific data availability from TCGA and GTEx, generating a unique (GTEx/TCGA) combined dataset when data from both resources were present. A comprehensive list of all processed tissues and the amounts of tissue-specific samples is reported in Supplementary Table S1.

### RNA-seq data from TCGA and GTEx projects

Tissue-specific RNA-seq data from either TCGA normal (non-tumor) samples or GTEx samples were downloaded from the Recount2 (28) project data portal and processed as follows: raw count matrices were extracted and filtered to retain only protein-coding genes according to GRCh38-v25 Human Gencode annotation (www.gencodegenes.org). Tissue-specific TCGA and GTEx RNA-seq samples were combined into a unique matrix and genes having RPKM ≥0.5 in at least the 10% of the samples were considered as expressed and hence retained in the downstream analyses. Normalized gene counts were then obtained using edgeR (29) followed by a voom-quantile normalization function (30) to correct for both technical and biological variability across samples. Tissue-specific TCGA and GTEx combined data was further normalized using ComBat (31) to adjust for the source-specific batch effect generated in the merging step.

### Genotype data from TCGA and GTEx projects

Tissue-specific raw TCGA genotype calls were downloaded from TCGA legacy data portal (portal.gdc.cancer.gov/legacy-archive) and converted into the common PLINK (32) file format (MAP/PED) retaining only genotypes with a score lower than 0.1. PED files underwent a first pre-filtering step to remove duplicate SNPs and discard variants with a call rate smaller than 0.75. MAP/PED files were then converted into more readable GEN/SAMPLE format using Gtool (well.ox.ac.uk/∼cfreeman/software/gwas/gtool.html). Chromosome-separated GEN files were then analysed with SHAPEIT v2 (33) to infer haplotype structure and optimize genotype content information for the imputation process. Variants were imputed using IMPUTE v2.3.2 (34) against a reference panel built from 1000 Genomes Project data. Imputed TCGA genotype calls were intersected with imputed GTEx genotype data obtained from dbgap (phs000424.p7.v2) and samples with overall call rate <0.9 were excluded. Only variants with MAF greater or equal than 1% were finally retained.

### Ancestry analysis

Ancestry analysis was performed using EthSEQ (35). For each tissue-specific TCGA/GTEx integrated genotype data, a selection of random 10% common variants with MAF >5% (about 700 000) were selected and used to run EthSEQ using a reference model built from 1000 Genomes Project data. The first three principal components of the PCA analysis performed by EthSEQ, which effectively describe the major populations structure (36), were extracted from EthSEQ results.

### Association of genetic variants genotype with transcript levels

Tissue-specific associations between variants genotypes and genes transcripts were calculated using the following model of linear correlation:

$$\begin{equation*} {\rm E} \sim \beta _0 + \beta _1 {\rm G} + \beta _2 {\rm A} + \beta _3 {\rm S} + \beta _4 {\rm PC1} + \beta _5 {\rm PC2} + \beta _6 {\rm PC3} \end{equation*}$$

where E is the transcript level of a gene, ${\beta _0}$ is the intercept coefficient, G is the genotype of a genetic variant, A is the individual's age, S is the individual's sex and PC1, PC2 and PC3 are the first three EthSEQ principal components. Each genetic variant was tested against all the genes expressed in the tested tissue using three different association models: additive, dominant, and recessive. In the additive model we grouped samples in three different genotype classes: reference homozygous, heterozygous and alternative homozygous. In the dominant model we combined the heterozygous samples with alternative homozygous while in the recessive model heterozygous are combined with reference homozygous. Age, sex and the three PCA terms were included to correct biases toward genes whose expression changes during life, variants that are more common in a sex with to respect to the other and effects on transcript that are due to individuals’ ancestry. We tested a variant for the association only if the genotype had at least 3 samples in each genotype class. *P*-values for the associations were obtained by a two-tailed t-test on the genotype coefficient ${\beta _1}$ under the null hypothesis that ${\beta _1}$ is equal to zero. For each variant and model, *P*-values were corrected using Benjamini-Hochberg method considering all tested genes as multiple hypothesis. Only associations with a corrected *P*-value less than 0.005 were considered for further analysis.

### Variants similarity network

Given a variant $v$, let $G$ be the set of genes annotated in Polympact having transcript levels associated with $v$. Now, let ${I_v}$ be the set of pairs such that:

$$\begin{equation*}{I_v} \subseteq G \times \left\{ { + , - } \right\}\end{equation*}$$

where a gene *g* is associated with ‘+’ when the variant alternative allele increases the transcript level of *g* and with ‘−’ when the variant alternative allele decreases it. Now, the similarity of two variants ${v_1}$ and ${v_2}$ in terms of transcript level associations (denoted also as *variantstranscripts similarity*) is defined as:

$$\begin{equation*}{S_{transcripts}}\ \left( {{v_1},{v_2}} \right) = \ Jaccard\ \left( {{I_{{v_1}}},{I_{{v_2}}}} \right) = \ \frac{{\left| {{I_{{v_1}}} \cap {I_{{v_2}}}} \right|}}{{\left| {{I_{{v_1}}} \cup {I_{{v_2}}}} \right|}}\end{equation*}$$

The function ${S_{motifs}}( {{v_1},{v_2}} )$ is defined by applying the same idea to binding motif alteration Polympact data (*variants motifs similarity*). Specifically, a gene is associated with ‘+’ when the variant alternative allele increases the binding motif score and with ‘−’ when the variant alternative allele decreases it.

Based on these definitions, we can define a similarity network where the nodes are variants and two variants are connected if and only if their transcripts (or motifs) similarity is greater than zero. Since connected variants may have relationships with common genes, we can use community detection algorithms to identify groups of variants presenting similar functional impact.

To study the similarity degree of variants’ pairs, we selected for each tissue all variants associated with the transcript level of at least one gene. Then, we computed with PLINK the sets of variants that are not in linkage disequilibrium using a genomic window of size 250kb and using 0.1, 0.5 and 0.8 as *r*^2^ thresholds. Finally, for each threshold, we built both variants transcripts and variants motifs similarity networks.

### Variant-gene network

Combining all data available in Polympact, we finally developed a model to describe the complex interaction landscape between a set of common genetic variants and genes. We formalized this model as a variant–gene network, defined as a directed bipartite graph where nodes are variants or genes, and edges are relations we found between variants and genes. Edges have a variant to a gene direction when the variant associates to the transcript level of the gene, while an edge has a gene to a variant direction when the gene binds at the variant locus.

To analyse Polympact variant–gene networks, we constructed for each tissue the network using all variants associated with at least one transcript level. Then, we analysed the networks structures by finding the strongly connected components and exploring centrality measures like degree, betweenness and closeness. Finally, we enumerated every possible 2-cycle in the network. All the analyses were conducted using NetworKit (37).

### Polympact database and web interface implementation

Polympact database is hosted on a MySQL version 5.7 containerized with singularity version 3.4. The web interface is implemented in Python3 using the Django framework version 3.0.5. The data visualization is obtained using Plotly-Dash version 3.1 for the heatmaps and Cytoscape-js 0.1.1 for the networks. Community detection in similarity networks is performed using the Louvain algorithm (38).

## RESULTS

### Overview of Polympact data

**Figure 1.**
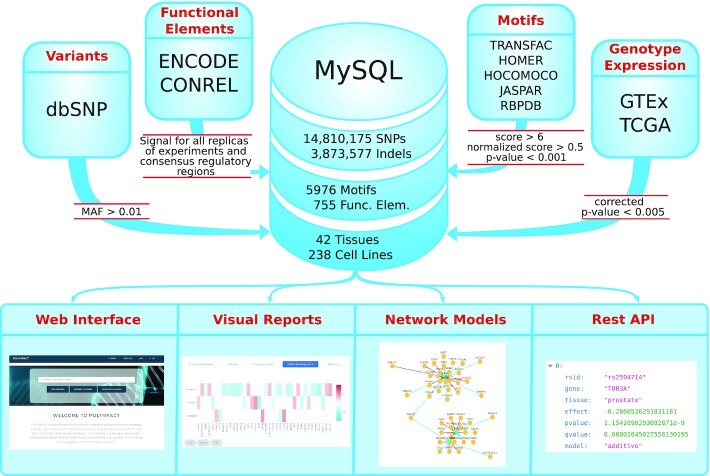
Polympact data and services. Polympact is implemented integrating common variants information and genotypes, ChIP-seq data, TFBS and RBP motifs and genotype/transcript level data retrieved and integrated from several databases. Data are filtered for high quality characteristics and stored in a MySQL database. Polympact offers an intuitive web interface providing visual reports and an innovative network visualization. It is also accessible programmatically through a REST API.

**Figure 2.**
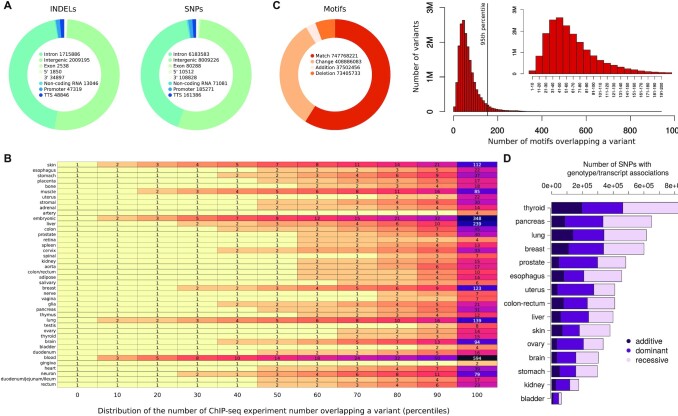
Summary of the data contained in Polympact. (**A**) Annotations for the different types of variants stored in Polympact. (**B**) Percentiles of the distribution of the number of variants overlapping a ChIP-seq peak in various tissues. (**C**) Types of binding motifs results and distribution of the number of motifs overlapping a variant for match, change, addition and deletion types. In small, a zoom of the major distribution part. (**D**) Number of variants associated with a transcript level in additive, dominant and recessive models in different tissues.

Polympact characterizes (Figure 1) more than 18 million common human genetic variants and allows for the exploration of: (i) their functional properties, exploiting more than 10 000 cell lines and tissue ChIP-seq experiments; (ii) their impact on binding motifs scores, exploiting about 6000 TFBS/RBP consensus motifs; (iii) their tissue-specific association with transcript levels, exploiting genotype and RNA-seq data of >5000 human individuals. A summary of the data contained in Polympact is reported in Figure 2.

More than 95% of the variants characterized by Polympact are non-coding and annotated as intergenic or intronic variants (Figure 2A). Specifically, 143 725 are annotated as variants in the 3′ UTR, 12 362 in the 5′ UTR, 10 018 421 are intergenic, 210 232 are located in a transcription termination site, 82,826 are exonic, 7 899 469 are intronic, 84 127 in non-coding RNAs and 232 590 in promoters.

Regarding the functional characterization of the variants, we found that 18 545 354 of 18 683 752 (99.26%) fall within at least one ChIP-seq peak (18 485 601 fall in at least one histone mark peak and 18 409 488 in at least one TF peak considering both broad and narrow peak data) in at least one tissue. As shown in Figure 2B, the majority of variants fall within few peaks across all tissues with half variants falling in two to three peaks in every tissue. In addition, 170 239 (0.9%) variants have a promoter annotation in at least one cell-line/tissue, whereas 7 839 972 (42%) have an enhancer annotation and 4 357 136 (23%) have an active enhancer annotation.

With respect to the TFBS motifs analysis landscape, we observed that >99.9% (18.678.853) of the variants cause at least one putative change, addition or deletion of a transcription factor. More specifically, 17 277 379 variants cause at least a putative change, 7 724 608 cause at least one addition and 8 859 076 cause at least one deletion. About 59% of the motifs analysis results are annotated as match, while 32% show a change in the score. Additions and deletions account, respectively, for the 3% and 6% of the overall motifs analysis results (Figure 2C, left). The distribution of the number of variants matching or altering a certain number of motifs show that we have >2.5 million variants overlapping 40−50 putative motifs that are annotated as matches, changes, additions or deletions. The distribution is slightly asymmetrical with very few variants that are associated with only 1 to 10 motifs (Figure 2C right). Putative change, addition or deletion of RBP motifs was observed in 95 265 UTR variants (66%), with 66 654 variants causing at least a putative change, 17 488 causing at least one addition and 27 412 causing at least one deletion.

Moving to the association with transcript levels, unlike eQTL analysis and similarly to what we have previously proposed in (24), association of genetic variant genotypes and transcript levels is here performed by testing each variant against all protein-coding transcripts, to search for association patterns that might be similarly shared across different variants. We found 3 653 655 variants with a total of 6 451 090 associations across fifteen tissues and three association models. Of these, 1 037 712 were additive associations, 2 555 425 were dominant and 2 857 953 were recessive. As shown in Figure 2D, thyroid was the tissue with the highest number of variants displaying associations (*N* = 873 525) and bladder the one with the lowest number (*N* = 63 448). Although the median value of associations per variant is one, the mean value is pretty variable across tissues (minimum 5.6 for uterus and maximum 104.2 for skin) indicating the presence of variants strongly enriched for associations. Indeed, as shown in Supplementary Table S2, while the 75th percentile of the tissue-specific variants associations distributions indicates an average number of associations per variant that is <3, when considering the 95th percentile, we observe an average value of 97 associations, which rapidly increases to 1171 associations when we consider the 99th percentile of the tissue-specific variants association distributions. Skin was the tissue with the highest number of total identified associations, while uterus was the tissue with the lowest number (Supplementary Figure S2). As expected, most (∼99%) of these associations are putative *trans* associations. Although our approach differs from standard eQTL analysis, we checked to what extent the putative *cis* associations we found are similar to the landscape of *cis* associations reported by GTEx eQTL data. Focusing for simplicity on a subset of tissues, we took the intersection between the variants characterized in GTEx and Polympact, and computed the fraction of *cis* associations in Polympact by selecting, similarly to GTEx, variants within one megabase of distance from the modulated gene in the selected tissues. We found a good concordance with about 60% of our *cis* associations that are also reported in GTEx and preserving in all cases the association direction (Supplementary Figure S3).

### Database and web interface

Polympact offers a web interface accessible through a web browser that can be used to query the resource by selecting the variants of interest and the preferred parameters setting. The only mandatory parameter is the list of variants IDs (in the form of rsids or strings with the variant position, reference and alternative alleles) while all the others are optional. The resource offers two search modes: quick and advanced search for both similarity and interaction analysis modes. The quick search is available from the home page (Supplementary Figure S4A) and retrieves the data for the requested variants using the default parameters (all tissues, all motifs effects and all associations models). In the advanced search page (Supplementary Figure S4B) a selection tree can be used to select a specific tissue of interest or a selection of specific cell lines available for that tissue. Using checkboxes, it is possible to specify the peak file format for the ChIP-seq data (narrow and/or broad peaks), the model used in the genotype/transcript association analysis (additive, dominant and/or recessive models) and the type of binding motifs results (match, change, addition and/or deletion). In addition, the corrected *P*-value threshold for the genotype/transcript association analysis (default 0.005) and the normalized difference in binding motifs scores (default 0) can be set to further filter displayed genetic variants results. Of note, only for a subset of selectable tissues the genotype/transcript association analysis data is available and the cell-line selection is exploited only for the analysis of functional elements.

**Figure 3.**
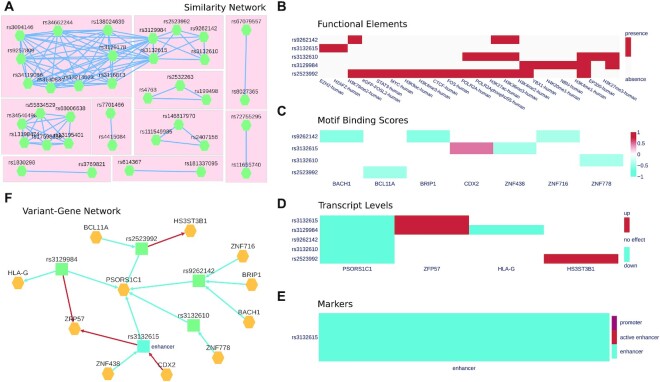
Polympact web interface. (**A**) Polympact similarity network model built from transcript association data. Each node represents a variant and two variants are connected if and only if they have a similarity greater than zero. (**B**) Histone marks and transcription factor ChIP-seq peaks overlapping in the genomic region of the variant. (**C**) Binding motifs scores. Red annotates variants that are increasing the binding score of the motif while blue annotates variants reducing it. (**D**) Genotype to transcript level associations. Alternative alleles lowering the transcript level are depicted in blue while alternative alleles increasing it are depicted in red. (**E**) CONREL marker annotations in the genomic region of the variant. (**F**) Polympact variant–gene network model. Genes are coloured in yellow while variants are in blue if they are annotated as putative enhancers or in green otherwise. Edges from a variant to a gene represents an association to transcript levels and are blue if the transcript are reduced and red if transcript levels are increased. Edges from a gene to a variant represent binding motifs changes and are red if the binding score is increased and blue if it is decreased.

Polympact similarity analysis provides an interactive interface to explore the similarity network of queried variants’ effects on transcripts levels (Figure 3A) or on binding motifs scores. Computed network communities are highlighted and each single community can be selected to perform an in-depth interaction analysis.

Polympact interaction analysis provides first a graphical representation, in the form of a heatmap, to explore functional relationships among the queried variants, separately for the functional elements, the binding motifs score alterations, and the transcript level associations. The heatmaps are accessible through, respectively, the ‘Functional Elements’, ‘Transcript Levels’ and ‘Motif Binding Scores’ tabs (Figure 3B–D) and are clustered using hierarchical clustering in a way that variants with similar characteristics are represented closer in the visualization. All the data is also reported in a tabular form and can be filtered and downloaded in various file formats. The ‘Markers’ (Figure 3E) tab provides additional insights into the regulatory role of the genomic regions spanning the variants and highlights links to our external resource CONREL to visualize the variant and its genomic context into a genome browser view. The variant–gene network model is accessible from the ‘Network’ tab (Figure 3F) where genes are reported in yellow and variants have colours representing their functional marker annotations across the cell lines/tissues selected. Edges are red if the variant alternative allele increases the binding motif score or is associated with increased transcript level; they are blue if the variant alternative allele decreases the binding motif score or is associated with decreased transcript level.

### Properties of similarity networks

**Figure 4.**
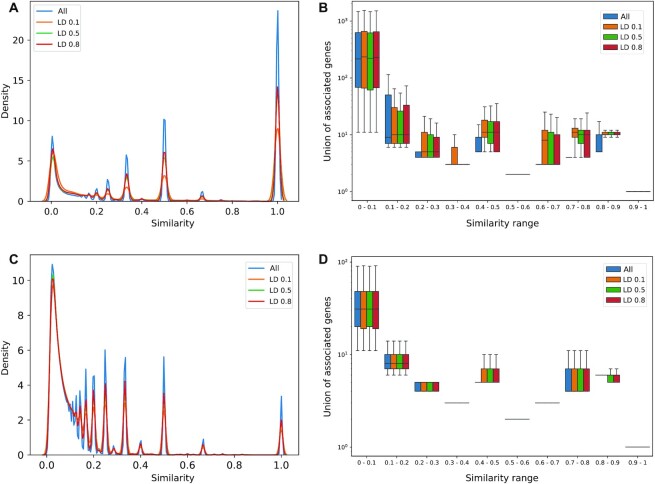
Analysis of variants similarity networks. (**A**) Distribution of variants similarity values in the breast transcripts similarity network. (**B**) Cardinality of the union of associated transcripts for each interacting pair stratified by similarity range and LD filtering. (**C**) Same as (A) but with variants motifs similarity values. (**D**) Same as (B) but with variants motifs similarity values.

Using Polympact data, similarity networks considering all variants’ pairs were created for 15 tissues. Networks based on binding motifs scores focused only on effects classified as addition or deletion, considered more relevant from a functional perspective. On average 1.5% (*N* = 1 241 691 365) of all possible variants’ pairs had a positive ${S_{transcripts}}$ similarity and 0.4% (*N* = 367 267 669) had a positive ${S_{motifs}}$ similarity. Comparable results were obtained when high linkage disequilibrium (LD) variants were filtered (Supplementary Table S3 and S4). Analysis of similarity values distributions across the networks revealed specific properties. Focusing for example on the breast tissue transcripts similarity network, but comparably for all other tissues, the distribution of ${S_{transcripts}}$ values was multimodal with a range of peaks located across the overall range [0,1] and the highest peak located in value one, representing perfect similarity (Figure 4A). As shown in Figure 4B, most similarities located in the highest peak were, as expected, from variants’ pairs associated with a single gene; in spite of that, we observed a tail of pairs involving tens of genes. Concordant distributions were obtained when correcting for linkage disequilibrium, demonstrating that a large fraction of similarities are not due to LD. Results obtained considering the distribution of ${S_{motifs}}$ similarity values were comparable (Figure 4C, D), further demonstrating the presence of a vast range of variants’ pairs not in LD sharing common functional relations.

### Properties of variant–gene networks

**Figure 5.**
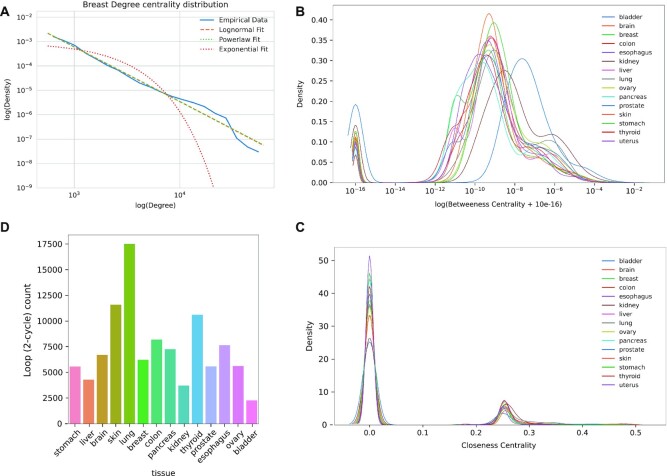
Analysis of variant–gene networks. (**A**) Log–log plot of the degree centrality distribution of the breast variant–gene network. (**B**) Betweenness centrality distribution of the variant–gene network across tissues. (**C**) Closeness centrality distribution of the variant–gene network across tissues. (**D**) Number of 2-cycles across tissues.

For each tissue, we created a variant–gene network considering all the variants associated with at least one transcript level in that tissue, and studied the topology of the network by exploring the number of connected components, the distribution of centrality metrics and the embedded 2-cycles. Each tissue network showed a similar topology, consisting of a single strongly connected component and many isolated nodes (Supplementary Table S5). Degree centrality distributions highlighted across all tissues a heavy tailed power law or a log-normal distribution with a likelihood ratio test propending for the log-normal distribution (Figure 5A, Supplementary Figure S5). Betweenness centrality distribution showed instead that, for each tissue, a large number of nodes do not participate in the network connections being the nodes outside the strongly connected component (Figure 5B). In particular, most tissues follow a similar distribution suggesting a conserved topological structure with bladder tissue showing a shift in the distribution, probably due to the low number of nodes in the network. Inspection of closeness centrality also showed a conserved distribution among tissues with a peak in zero, where all the nodes not belonging to the strongly connected component are located, and a second peak near 0.25, which contains the nodes in the main connected component (Figure 5C).

We then focused on variant gene network cycles, which are structures involving relations between variants and genes. Specifically, we focused on variants associated with the transcript level of a TF that are also modifying the binding motif score of the same TF, forming a 2-cycle in the network. Cycles are of particular interest because they may underlie the presence of positive or negative feedback loops between variants and transcription factors. We observed 2-cycles in every tissue (Figure 5D) from a maximum of 17 522 in lung to a minimum of 2283 in bladder. By investigating the possible functional impact of 2-cyles we found that variants involved in 2-cycles are enriched in functional markers (*P*-value = 1.7e−76, Supplementary Table S6).

### Case studies

To explore the utility of Polympact, we considered a first case study based on cancer risk GWAS common variants and a second case study based on Alzheimer's disease risk GWAS common variants (Supplementary Tables S7 and S8).

### Cancer risk GWAS variants

2657 variants related to cancer were retrieved from the GWAS catalogue (1), of which 2370 were present in Polympact.

We first explored the landscape of functional annotations across the loci identified by the GWAS risk variants. After computing the extent of linkage disequilibrium across the 2370 variants using the Ensembl REST API (39), we identified 1958 LD blocks; we considered pairs of variants with an *r*^2^ >0.5 to be LD. Then we built 100 random GWAS sets where a single variant is randomly selected from each LD block, hence obtaining 100 sets of 1,958 GWAS variants that are not in LD. We also created 100 sets of 1958 random variants selected among all variants in Polympact (excluding the 2370 GWAS variants) and preserving the distribution of minor allele frequency of the GWAS variants.

**Figure 6.**
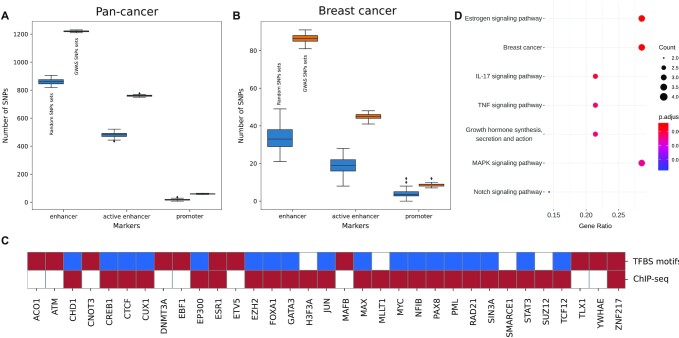
Pan-cancer and breast cancer GWAS analysis. (**A**) GWAS variants associated to all cancer types annotated as enhancers, active enhancers and promoters compared with random sets of variants. (**B**) Same as A) but with GWAS variants associated to breast cancer risk. (**C**) Cancer related TFs over-represented in breast cancers GWAS variants with respect to random sets in ChIP-seq data and TFBS motif alterations. Red *P*-values <0.01, blue *P*-values >0.01, white no data. (**D**) Cancer related terms resulting from the gene set enrichment analysis on all transcription factors over-represented for ChIP-seq and with TFBS motif alterations.

We then counted for each GWAS and random variant the number of overlapping marker regions and compared the distribution of counts in the GWAS variants sets with respect to the random variants sets. As shown in Figure 6A and Supplementary Figure S6A, markers of promoters, enhancers, active enhancers together with a subset of histone marks result enriched in the GWAS sets with respect to the random sets (*P*-value < 0.01), clearly supporting the observation that variants associated with cancer risk have an active functional role.

Being the number of risk variants reported in the GWAS catalogue not uniformly distributed across the different cancer types, we decided to further explore risk variants functional properties by focusing only on a single cancer type. Specifically, we selected the richest subset of 853 GWAS variants that are associated to breast cancer risk, 808 of which are characterized in Polympact. Of those, 58 variants are associated with at least one transcript level with 445 total unique associations, of which 71 (∼16%) are *cis*-associations. Out of the 808 variants, we identified 653 LD blocks and we generated as previously 100 random sets of 653 GWAS variants (not in LD) and 100 random sets of 653 random variants. Also in this case, markers of promoters, enhancers, active enhancers and a selection of histone marks resulted enriched in the GWAS variants sets with respect to the random variants sets (*P*-value < 0.01, Figure 6B and Supplementary Figure S6B). In addition, more than 30 genes known to be implicated in cancer were found to have enriched transcription factor functional peaks in the GWAS variants sets with respect to the random variants sets and/or binding motifs that are changed, added or deleted by GWAS variants alternative alleles (Figure 6C). In particular, the estrogen receptor *ESR1* and the oncogene *ZNF217* are both enriched for functional peaks in the GWAS variants and have binding motifs that are significantly impacted by a subset of the same variants. Interestingly, focusing more generally on all transcription factors (not only cancer genes) that have this dual characteristic, we found a set of genes that enrich (40) for pathways related to hormone synthesis, estrogen signalling and breast cancer (Figure 6D), overall supporting the implication of breast cancer GWAS variants in cancer relevant biological processes.

**Figure 7.**
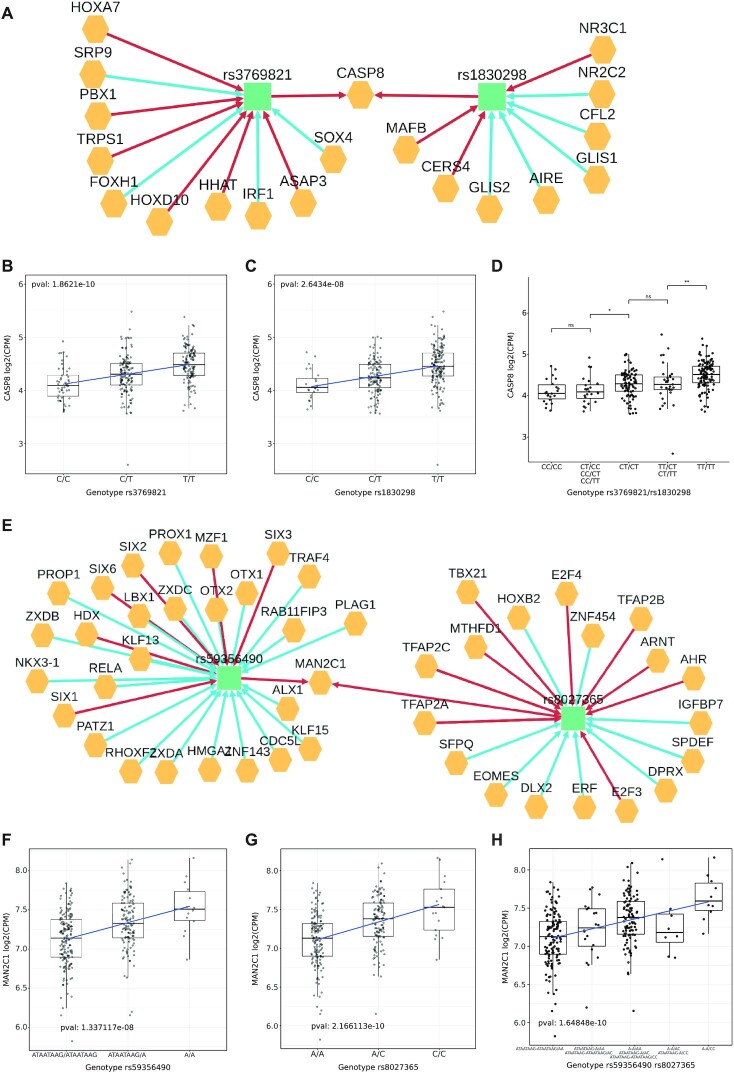
Effect of variants on *CASP8* and *MAN2C1* transcripts. (**A**) Variant-gene network of the two variants rs3769821 and rs1830298. (**B**) Effect of the variant rs3769821 on the transcript level of *CASP8* gene under the additive model. (**C**) Effect of the variant rs1830298 on the transcript level of *CASP8* gene under the additive model. (**D**) Combined effect of the two variants rs3769821 (first pair of nucleotides in the label) and rs1830298 (second pair) on *CASP8*. (**E**) Variant-gene network of the two variants rs59356490 and rs8027365. (**F**) Effect of the variant rs59356490 on the transcript level of *MAN2C1* under the additive model. (**G**) Effect of the variant rs8027365 on the transcript level of *MAN2C1* under the additive model. (**H**) Combined effect of the two variants rs59356490 (first pair of nucleotides in the label) and rs8027365 (second pair) on *MAN2C1*.

To further characterize the functional role of breast cancer risk GWAS we analysed all the 808 variants using the Polympact transcripts similarity network created with standard parameters and focusing on breast tissue. We found 10 unique network communities (Figure 3A). Out of them, we selected 4 communities associated with genes *CASP8*, *MAN2C1*, *BTN3A2* and *ARL17A* which are all reported in literature as possibly involved in cancer. The *CASP8* network community contains the two variants rs1830298 and rs3769821. In particular, the variant rs1830298 is 60 kb far away from the variant rs3769821, which is annotated as an intron variant of *CASP8*. Variant rs1830298 alternative allele reduces the binding score of *NR2C2* hormone receptor while rs3769821 decreases the binding score of the tumor suppressor *IRF1* (Figure 7A); both variants have the GWAS catalogue reported risk allele (allele C) that is strongly associated with a decrease in the *CASP8* transcript levels (Figure 7BC). Of note, our integrated TCGA and GTEx dataset from which the associations were computed is composed by individuals with mainly European (75%) and African (16%) ancestry, populations were the two variants have respectively moderate (*r*^2^ ∼ 0.5) and low (*r*^2^ ∼ 0.2) linkage disequilibrium. Inspection of transcript levels at all variants genotype combinations (Figure 7D) revealed that homozygous alternative genotype (TT genotype for both variants) is needed to sustain on average high *CASP8* transcript level. In particular, a first decrease of *CASP8* levels is observed in presence of the risk allele C in at least one variant (e.g. when at least one variant has heterozygous genotype) and a further decrease is observed when at least one variant has homozygous CC genotype. These results, together with the genotype combinations observed from phased data retrievable from the 1000 Genomes Project data (Supplementary Figure S7) strongly suggest a putative interaction effect that rs1830298 and rs3769821 have in maintaining a high level of *CASP8* transcript, which is lost in individuals carrying the breast cancer risk allele in at least one of the two variants.

The *MAN2C1* network community (Figure 7E) includes SNP rs8027365 (*PTPN9* intron variant, risk allele A) and the small deletion rs59356490 (intergenic variant, risk allele deletion not present) located 120 kb away and they are not reported to be in LD. Both variants modulate additively the transcript level of *MAN2C1* (Figure 7F−H) and variant rs59356490 has a functional annotation for *POLR2A* and *ESR1* and overall it deletes 23 TFBS motifs. The combination of the two variants shows an additive trend where the highest transcript level is reached when both variants are present.

The *BTN3A2* network community contains 6 variants: rs13195401, rs13198474, rs17598658, rs34546498, rs55834529 and rs68006638 and they are all associated with a decrease in transcript level for *BTN3A2* gene in the dominant model. Among them we selected the pair rs13195401 (annotated as *BTN2A1* non-sense variant, risk allele G) and rs13198474 (annotated as *SLC17A3* 5′ UTR variant, risk allele G) having the lowest LD (*r*^2^ = 0.49) in the general population (Supplementary Figure S8). The combination of the two effects shows a trend where the decrease is small when only the variant rs13195401 is present, the decrease is higher when only the variant rs13198474 is present, and the highest decrease in the transcript level is reached when both variants are present.

Finally, we analysed the network community of *ARL17A*. The two variants in this community are rs2532263 (*KANSL1* intron variant, risk allele G) and rs4763 (*ARHGAP27* 3′ UTR variant, risk allele G) and both are associated with an increase of *ARL17A*, *LRRC37A*. *LRRC37A2* and *CRHR1* genes transcript levels. For all genes the variants have a full additive effect similarly to *MAN2C1* (Supplementary Figure S9).

Interestingly, we also found that the variant rs8050871, located in a region annotated as active enhancer, has a *cis* effect on the transcript level of gene *ZNF23* causing a decrease in its transcript level. The variant also deletes a binding motif for the same TFs creating a loop (a 2-cycle) in the variant gene network. Overall, this suggests that the variant is possibly involved in a regulatory positive feedback loop, potentially inducing dynamic instability.

### Alzheimer's disease GWAS variants

1044 common variants related to Alzheimer's disease were retrieved from the GWAS catalogue, 810 of which were present in Polympact.

To highlight the utility of Polympact in identifying more putative functional relations, we analyzed the 810 variants exploiting the transcripts similarity network computed on the brain tissue. We identified 4 network communities and focused only on the 3 ones composed by variants that are not in high LD.

The first community contains variants rs199499 and rs7207400, that are reported in the GWAS catalogue as associated to trait *Alzheimer's disease in APOE ϵ4- carriers* and that present a low LD in the general population (*r*^2^ = 0.18) and moderate LD (*r*^2^ = 0.52) in the European population. The second community is formed by three variants, rs113260531, rs7225151 and rs80257887, that are reported as associated to trait *Alzheimer's disease or family history of Alzheimer's disease*; the first two variants are located on chromosome 17 and are in moderate LD (r^2^ = 0.67) while the third variant is located on chromosome 19.

Finally, the last community is formed by variants rs7963314 and rs79926713, associated with trait *Alzheimer's disease and Late-onset Alzheimer's disease* and are located on two different chromosomes.

**Figure 8.**
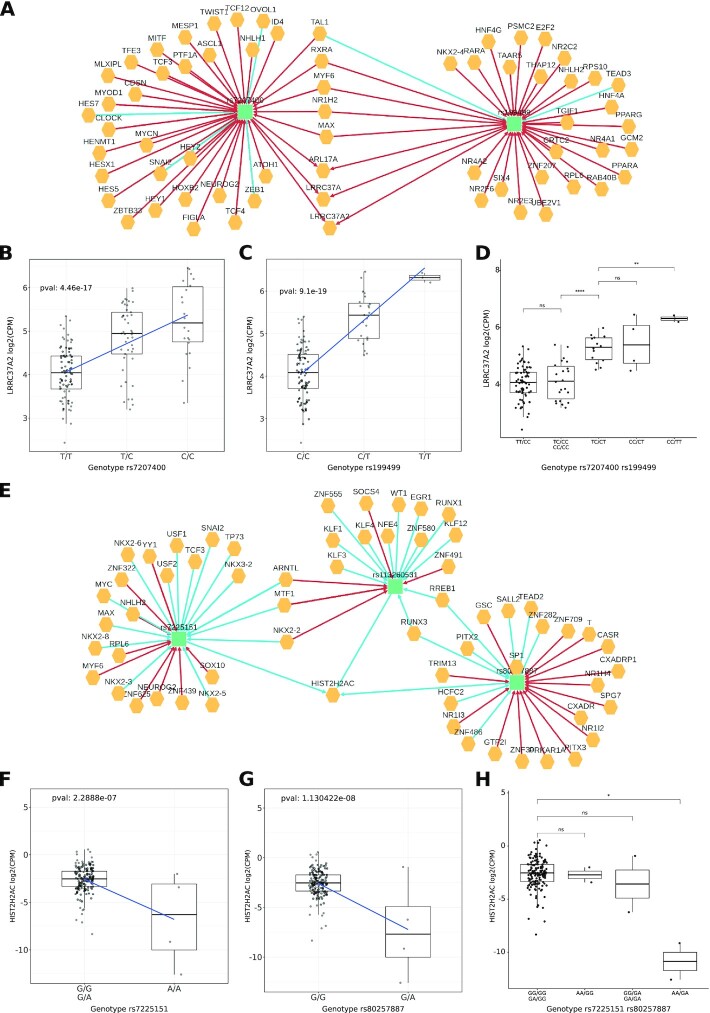
Alzheimer's disease GWAS variants analysis. (**A**) Variant-gene network of the two variants rs7207400 and rs199499. (**B**) Effect of the variant rs7207400 on the transcript level of *LRRC37A2* gene under the additive model. (**C**) Effect of the variant rs199499 on the transcript level of *LRRC37A2* gene under the additive model. (**D**) Combined effect of the two variants rs7207400 (first pair of nucleotides in the label) and rs199499 (second pair) on *LRRC37A2*. (**E**) Variant-gene network of the community formed by variants rs7225151, rs113260531 and rs80257887. (**F**) Effect of the variant rs7225151on the transcript level of *HIST2H2AC* gene under the recessive model. (**G**) Effect of the variant rs80257887 on the transcript level of *HIST2H2AC* gene under the dominant model. (**H**) Combined effect of the two variants rs7225151(first pair of nucleotides in the label) and rs80257887 (second pair) on *HIST2H2AC*.

The first community variants are located on chromosome 17 and are about 1Mbp afar. Variant rs199499 is annotated as intron variant of gene *LRRC37A2* and is located about 800kB downstream to the gene *MAPT*, while rs7207400 is annotated as intron variant of *LINC02210*-*CRHR1*. The Polympact network (Figure 8A) shows that both variants are increasing the transcript levels of *LRRC37A*, *LRRC37A2* and *ARL17A*. Interestingly, both variants are increasing the binding of MYF6 and have an opposite effect on the binding of TAL1. Also, rs7207400 creates new binding for MYCN and TCF4. Risk alleles (C for rs199499, T for rs7207400) are strongly associated with decreased levels of gene transcripts *LRRC37A*, *LRRC37A2* and *ARL17A* (Figure 8BC and Supplementary Figure S10ABDE). Genotype combinations (Figure 8D and Supplementary Figure S10C, F) show that absence of risk allele for both variants is needed to guarantee the highest transcripts levels. A first decrease in transcript levels is indeed observed when one of the two variants has heterozygous genotype and a second decrease is observed when one of the two variants has homozygous genotype for the risk allele. In addition, 1000 Genomes Project phased genotypes indicate that risk variants are almost always present on the same allele (Supplementary Figure S11). Overall, the data suggest that both rs199499 and rs7207400 non-risk alleles are required in phase to sustain the highest levels of *LRRC37A*, *LRRC37A2* and *ARL17A* transcripts.

The second community is composed by variants located on different chromosomes: chromosome 17 for variants rs113260531 and rs7225151 (both annotated as upstream variants for gene *SCIMP*, risk allele A) and chromosome 19 for variant rs80257887 (annotated as intron variant of *CEACAM20*, risk allele A). The Polympact variant–gene network (Figure 8E) shows that variants rs113260531 and rs80257887 are significantly decreasing the binding scores of RUNX3 and RREB1 and have alternative alleles associated with decreased *HIST2H2AC* gene transcript levels. We selected variants rs7225151 over rs113260531 having more alternative homozygous samples for further analysis. Specifically, a recessive effect is observed for variant rs7225151 (Figure 8F) with AA genotype associated with lower *HIST2H2AC* transcript level, while a dominant effect is observed for variant rs80257887 (Figure 8G) with AA or AG genotype associated with lower *HIST2H2AC* transcript level. Notably, a reduction of *HIST2H2AC* transcript level (Figure 8H) is evident in individuals carrying both AA risk genotype for variant rs113260531 and AA or AG risk genotype for variant rs80257887.

Finally, we analysed variants rs7963314 and rs79926713. Variant rs79926713 is located on chromosome 6 (annotated as intron variant of *SYNGAP1*, risk allele T) while rs7963314 is located on chromosome 12 (annotated as intergenic, risk allele A). Variant rs79926713 is annotated as promoter and is associated with an increase in transcript of gene *PPP1R12A* in the recessive models. Variants rs7963314 is instead associated in the modulation of 19 genes in the recessive model, including *PPP1R12A* gene (Supplementary Figure S10GHIJ).

## DISCUSSION

The study of common human genetic variants can provide insights into the biological cause of complex traits and diseases. Although several databases and web applications have been developed in the last decade to annotate and characterize genetic variants, the aggregation of these information to identify variants links and interactions has been largely unexplored. To this aim we developed Polympact, a tool that enables the exploration and the analysis of common genetic variants and their potential interactions by exploiting the integration of a large variety of biological data and analyses. Reasoning that variants’ interaction could be identified by characterizing their impact and involvement in the modulation of same genes or same biological pathways and processes, we first designed a workflow to uniformly characterize a large amount of common variants based on specific functional properties retrieved from well-known public databases. Then, on top of this uniform and homogenous annotations we developed a framework to represent and explore variants functional relations. More specifically, we combined genotype data together with transcription factor and histone marks ChIP-seq peak data, TFBS and RBP motifs data and transcriptomic profiling via RNA-seq across multiple human tissues, and we implemented a framework, provided as a dedicated web-server, to systematically characterize variants and to explore the landscape of variants functional relations through the combination of clustering analysis and novel network models.

While the uniform characterization of variants provided by Polympact was tailored with respect to the built clustering and network models, the resource we provide extends and complements annotations provided by other databases. Indeed, Polympact binding motifs data were determined both for an extended number of variants and an extended number/type of motifs. The recent SNP2TFBS tool (7), for example, characterizes only around 3 million SNPs and uses only Jaspar database (22). Of note, provided that variants in UTRs can alter mRNA translation potential (41) also RBP consensus motifs were included to characterize UTR variants. In addition, our functional characterization in terms of regulatory elements uses our recent CONREL tool (18), exploiting hence a novel tissue level functional annotation of variants. Further, our genotype/transcript association analysis approach well complements eQTL interaction data and was already proven successful in characterizing and prioritizing variants in terms of their impact on specific genes or biological processes (24). Although we recognize that this approach could limit the identification of moderate/weak *cis* effects, a good concordance with GTEx *cis*-eQTL data is shown, and overall we believe that enabling the identification of *trans* effects is fundamental to unravel key features of the architecture of complex diseases (42).

To characterize variants’ functional relations, we first introduced the notion of similarity network, which allows for the identification of variants that have common effects on the level of the same transcripts or the binding score of the same TFs/RBPs. In-depth analysis of the distribution of variants’ pairs similarities across networks built from different tissue data, revealed how these distributions are highly conserved also when keeping only variants not in linkage disequilibrium, supporting hence the presence of many independent variants that can possibly interact and further highlighting a landscape of complex patterns in gene regulation.

Additionally, we introduced the notion of variant–gene network, which provides a detailed network view of variants and genes interactions across different tissues integrating all Polympact data. In-depth analysis of these networks built from different tissue data, revealed heavy-tailed degree distribution highlighting the presence of regulatory hubs (variants or TFs) in the network. We also analyzed the 2-cycles present in the networks showing that variants forming these type of loops are enriched for regulatory markers, suggesting hence the possible presence of positive and negative feedback loops related to specific TFs. Overall, the analysis unravelled a complex topology and highlighted that our variant–gene network can be a useful tool to detect and analyse complex interaction patterns. Additional mesoscale and group-centric metrics could be considered to further explore properties of these large networks (43).

Using the exhaustive list of common genetic risk variants available from the GWAS catalog, we then showed that Polympact is able to highlight important features and functional relations among disease risk variants in terms of their functional genomic context, binding motifs alterations and transcript level modulations.

Exploiting Polympact data we first showed that cancer GWAS risk variants are enriched for regulatory elements annotations, in line with previous studies (44–46). In addition, we found that the set of transcription factors with functional peaks enriched for GWAS variants and having binding motifs modified by the same variants have a statistically significant role in cancer-related pathways, suggesting that GWAS variants may hence modulate downstream effects of oncogenic pathways. Specifically, focusing on GWAS breast cancer risk variants we found a set of transcription factors enriched in pathways specific to breast cancer and to response to hormone related pathways. This, in line with previous observations made by us (24) and others (47) in the context of other hormone driven cancers, suggests that common genetic variants may modulate downstream effects of hormone signalling by altering the binding of hormone receptors or hormone regulated genes, potentially favouring the risk of developing cancer in only a subset of individuals carrying a specific genetic makeup. Notably, our analysis highlighted *ESR1* (estrogen receptor alpha), *GATA3* gene which is known to influence response to estrogen (48) and the well-known oncogene *MYC*. In particular, our data shows that GWAS variants associated with breast cancer risk not only are enriched in regions that are bound by the estrogen receptor but also tend to alter the way in which ESR1 binds these regions.

Further inspection of the network models built by Polympact on breast cancer GWAS variants revealed a putative interaction between two variants that, when present on the same allele, synergistically modulate the transcript level of *CASP8* gene, a key regulator of apoptotic response already shown to be downregulated in breast cancer (49,50) and involved in cancer initiation when deficiently expressed (51,52). Specifically, we have shown that *CASP8* transcript level is reduced when GWAS risk allele for at least one of the two variants is present, with the lowest expression that is observed when at least one of the two variants has a risk allele homozygous genotype. This suggests that the presence of the GWAS risk allele may favour the evasion from apoptosis, a well-known cancer hallmark, increasing hence the risk of breast cancer initiation. Our findings are in line with (53) where the authors show that the strongest associations with breast cancer risk in the region come from variant rs1830298 and that variant rs3769821 is an eQTL for *CASP8*. Our results are consistent with the authors’ hypothesis that one or more variants in the region are responsible for the reduced expression in *CASP8*.

With respect to our results related with *MAN2C1* gene, it has been shown that the gene may inhibit the function of tumor suppressor gene *PTEN* in breast and prostate cancer (54) and another study found that the gene may have a protective role in cancer initiation with respect of progression (55). In our analysis, each risk allele of variants rs8027365 and rs67079557 contribute to a reduction in the expression of *MAN2C1* transcript, suggesting hence a protective role of *MAN2C1* in breast cancer initiation.

In the context of breast cancer GWAS variants we also found variant rs8050871, involved in a 2-cycle. The variant is located in a putative active enhancer and simultaneously associated with decreased transcript level of *ZNF23* and decreased *ZNF23* binding at the variant locus. Provided that *ZNF23* is a gene downregulated in cancer and associated to inhibition of cell-cycle progression (56,57), the identified feedback loop could potentially contribute to an enhanced cellular proliferation and potentially an increased cancer risk.

Searching for additional examples of multiple variants functional relations, we studied GWAS variants associated to Alzheimer's disease and showed that absence of risk alleles for variants rs199499 and rs7207400 is necessary to sustain the transcript level of several genes (*LRRC37A*, *LRRC37A2* and *ARL17A*) in the complex genomic region 17q21.31. This region, which hosts the Alzheimer related *MAPT* gene (58,59), is known to have undergone an inversion event during evolution (60) and to be associated with abnormal tau protein deposit (61). Both rs199499 and rs7207400 variants were observed to modify the binding motif of *TAL1* gene, which is known for its effects on GABAergic neurogenesis (62). Variant rs7207400 also creates a binding motif for the TCF4 transcription factor, involved in synaptic plasticity (63), and the well-known *MYCN* gene, essential in neurogenesis.

We also found that specific rs113260531, rs7225151 and rs80257887 variants risk allele patterns reduce the transcript level of *HIST2H2AC*, a histone protein shown to be downregulated in brain blood vessels of Alzheimer's disease mouse model (64). Variants rs7225151 and rs80257887 are in moderate LD (r2 = 0.6) while rs113260531 is located on a different chromosome. Variants rs113260531 and rs80257887 were also observed to decrease the binding score of RUNX3, a transcription factor that is essential in the development and fundamental formation of axons (65), and RREB1, a regulator of glutamatergic axons death (66).

Overall, we have shown that Polympact represents a useful tool to explore functional annotations and properties of common genetic variants, leading not only to an effective characterization of single variants but also to an effective investigation of putative functional relations and potential interactions among multiple variants. We hence believe Polympact might be broadly applied and used to generate hypothesis about the biological causes of complex diseases.

## DATA AVAILABILITY

Polympact is available at bcglab.cibio.unitn.it/polympact.

## Supplementary Material

gkac024_Supplemental_Files
