# Supplementary material for: Polympact: exploring functional relations among common human genetic variants
Source: Nucleic Acids Res. 2022 Jan 21;50(3):1335–50. doi: 10.1093/nar/gkac024 (PMC8860573; doi:10.1093/nar/gkac024)
Supplement: gkac024_Supplemental_Files [file gkac024_supplemental_files.zip › Supplementary_Figures.pdf]

SUPPLEMENTARY FIGURES

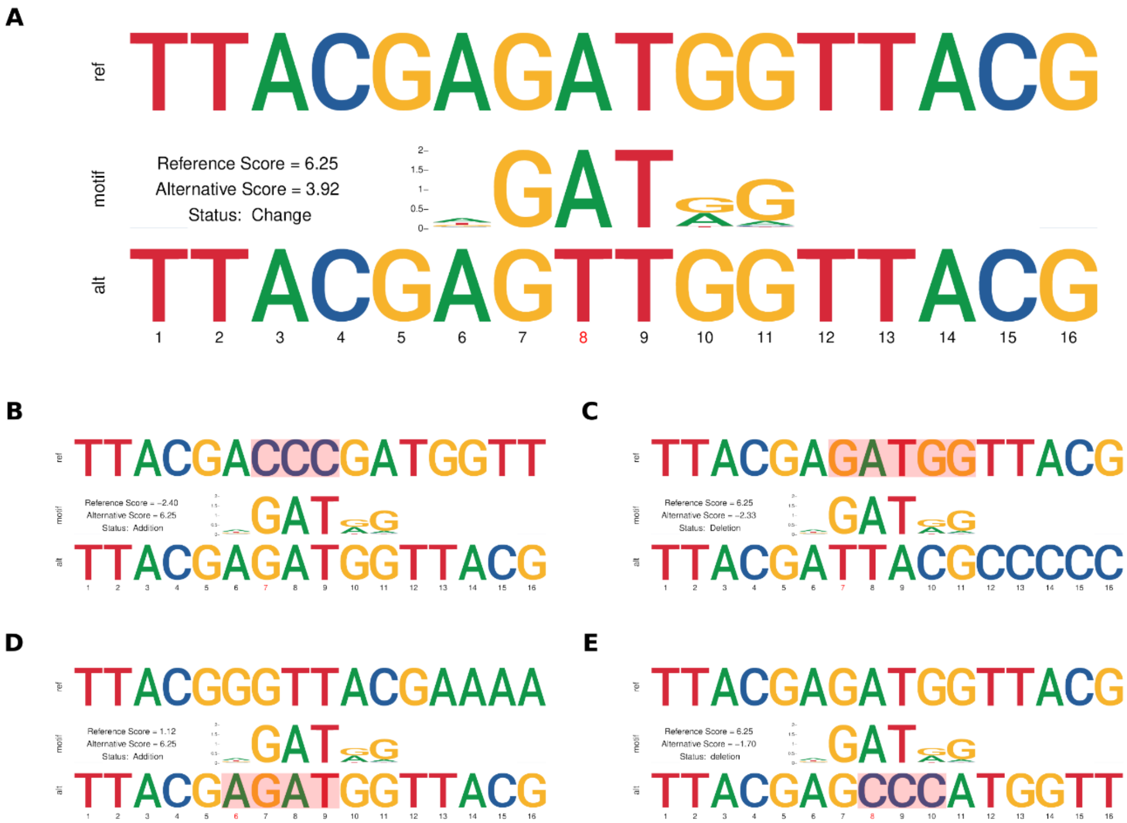

Figure S1. A) SNP alteration of a TFBS consensus motif in position 8 annotated as change since the difference in score is greater than 10%. **B)** Small deletion of the red nucleotides starting from position 7 annotated as motif addition since reference score is negative and alternative score is positive. **C)** Small deletion of the red nucleotides starting from position 7 annotated as motif deletion since reference score is positive and alternative score is negative. **D)** Small insertion of the red nucleotides starting from position 6 annotated as motif addition because the motif starts inside the insertion region. **E)** Small insertion of the red nucleotides starting from position 8 annotated as deletion because the reference score is positive and the alternative score is negative.

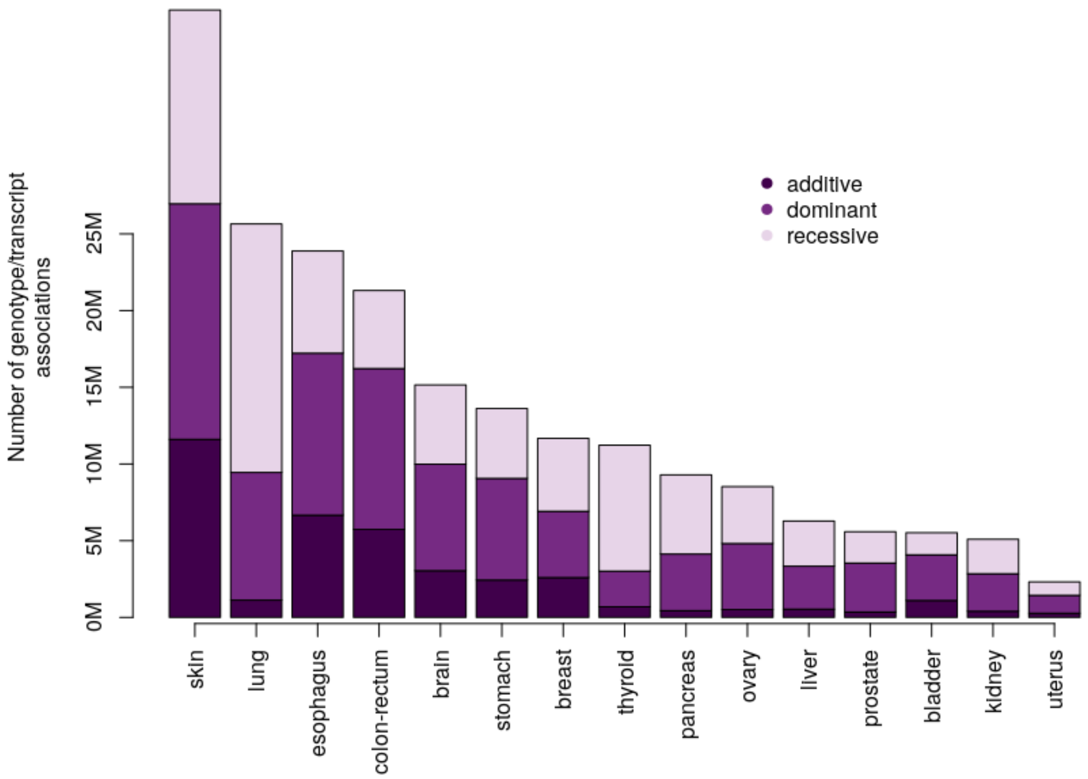

Figure S2. Total number of genotype/transcript associations in various tissues stratified by the three different association models.

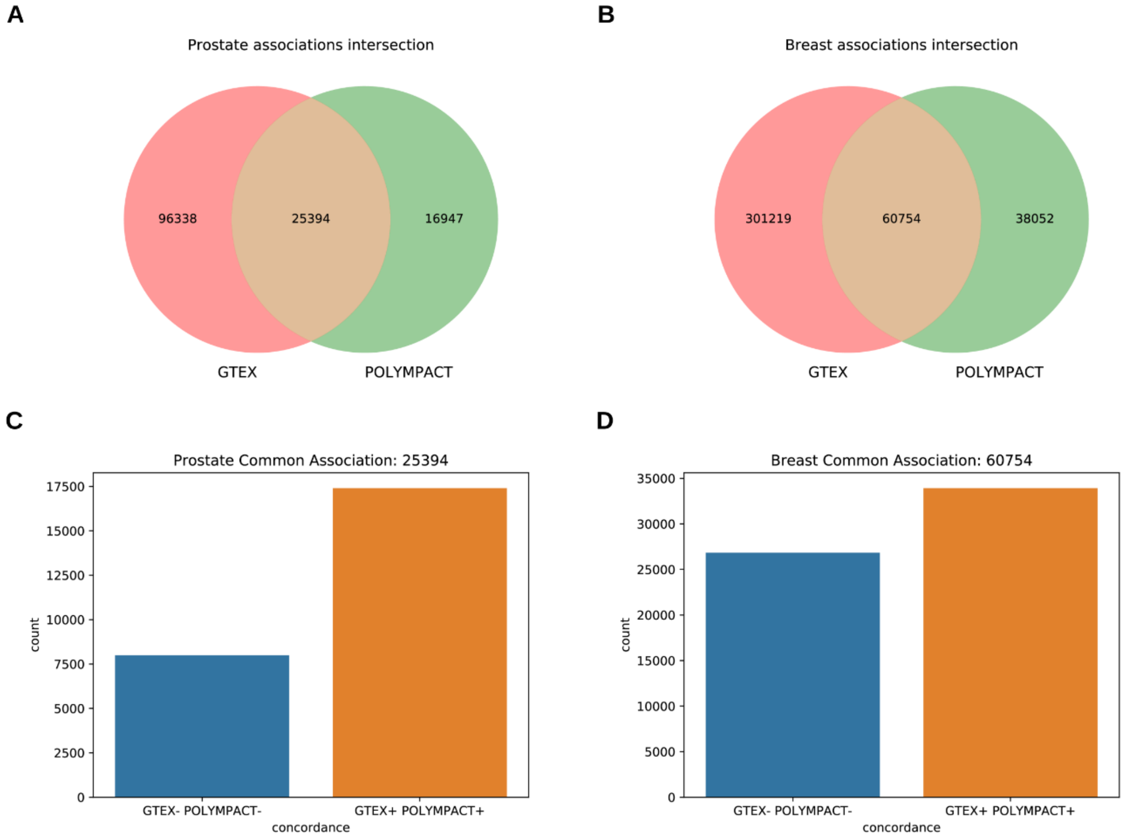

Figure S3. A) Venn diagram of Polympact and GTEx prostate tissue *cis* associations. **B)** Same as A) but considering breast tissue *cis* associations. **C)** Comparison of the direction of the associations in the prostate tissue data; GTeX- Polympact- represent associations with negative effect on the transcript levels in both GTeX and Polympact, while GTeX+ Polympact+ represent associations with positive effects. **D)** Same as C) but with breast data.

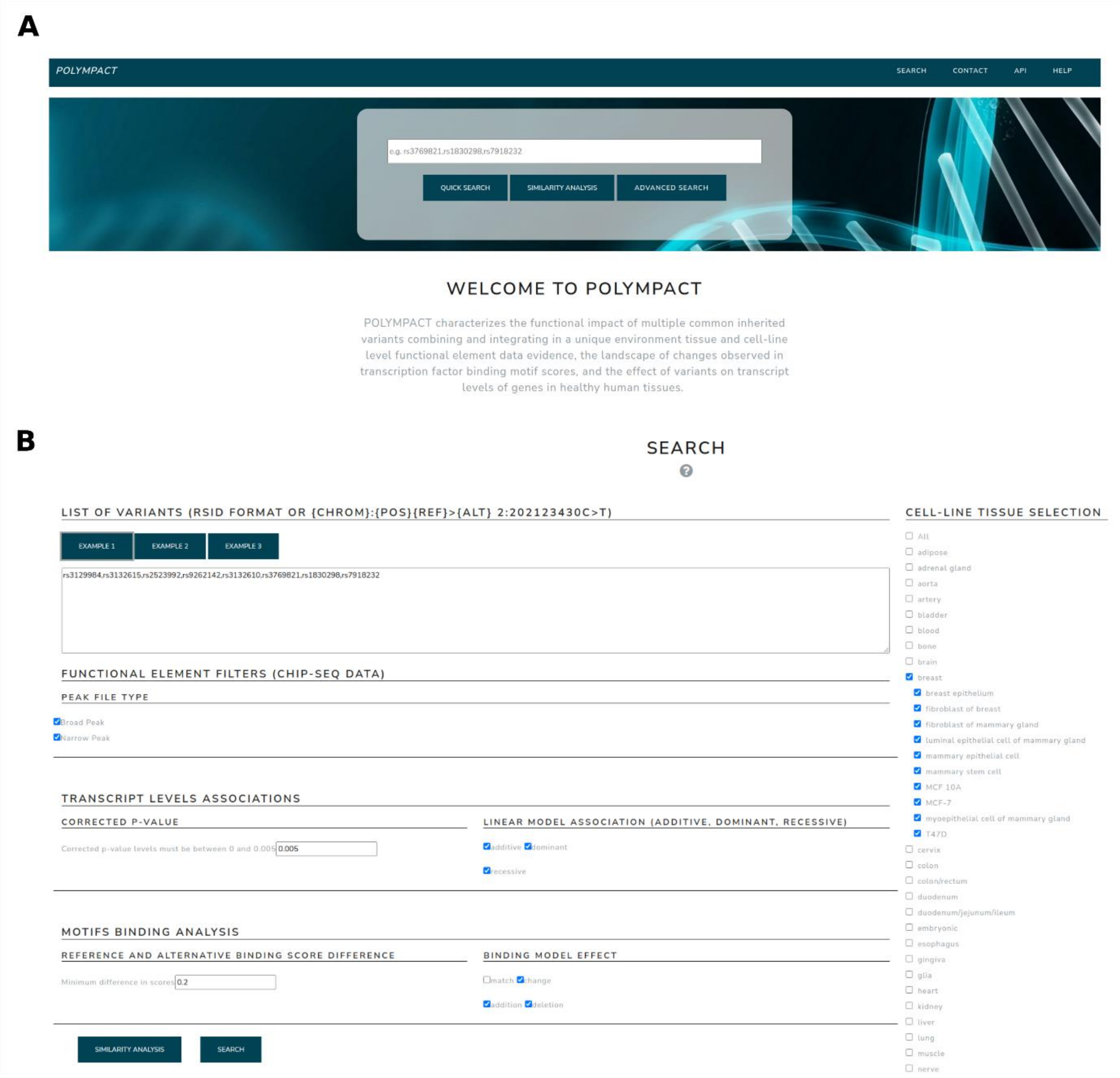

Figure S4. A) Quick search interface. The only required input are the variants provided in different formats and separated by commas. The search is performed using default parameters. **B)** Advanced search interface. The user can select specific tissues/cell lines, ChIP-seq peaks files types, transcript level association models and stringency and type of motif binding to refine the search.

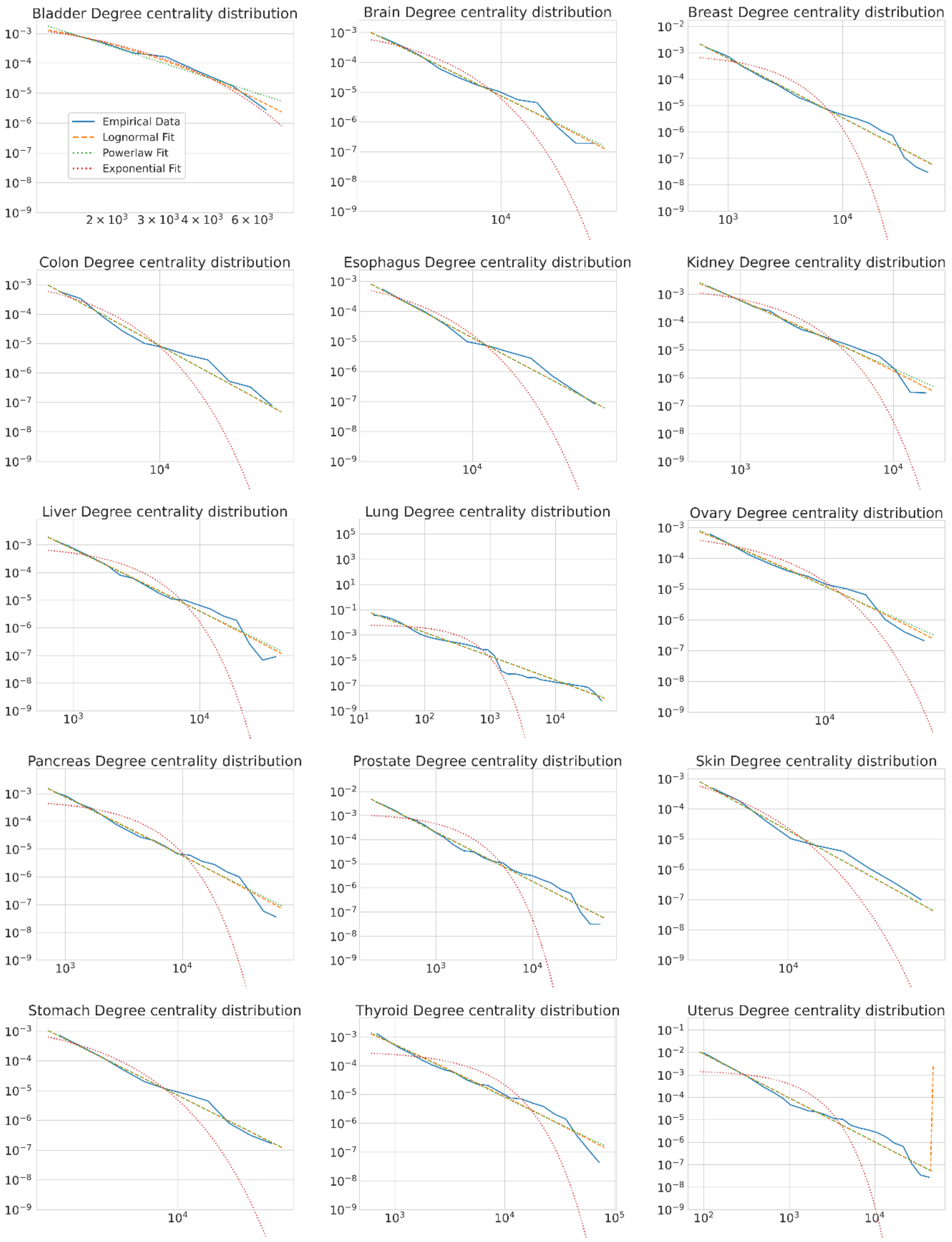

Figure S5. Degree centrality distributions of the variant-gene networks across all tissues analyzed in Polympact.

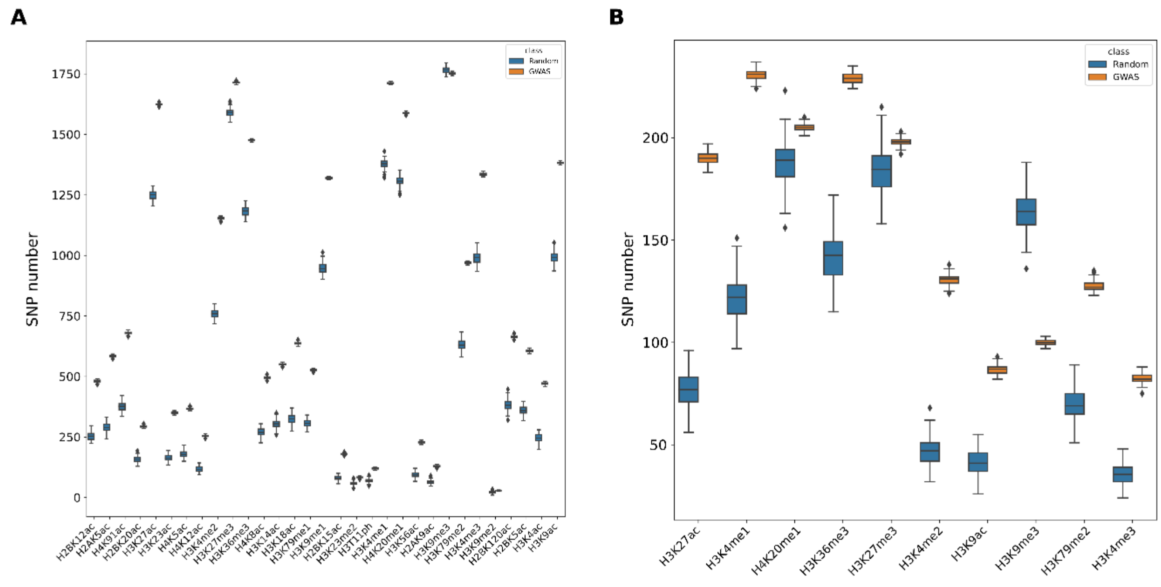

Figure S6. A) Histone marks enriched by GWAS cancer risk variants. **B)** Same as A) but considering GWAS breast cancer risk variants.

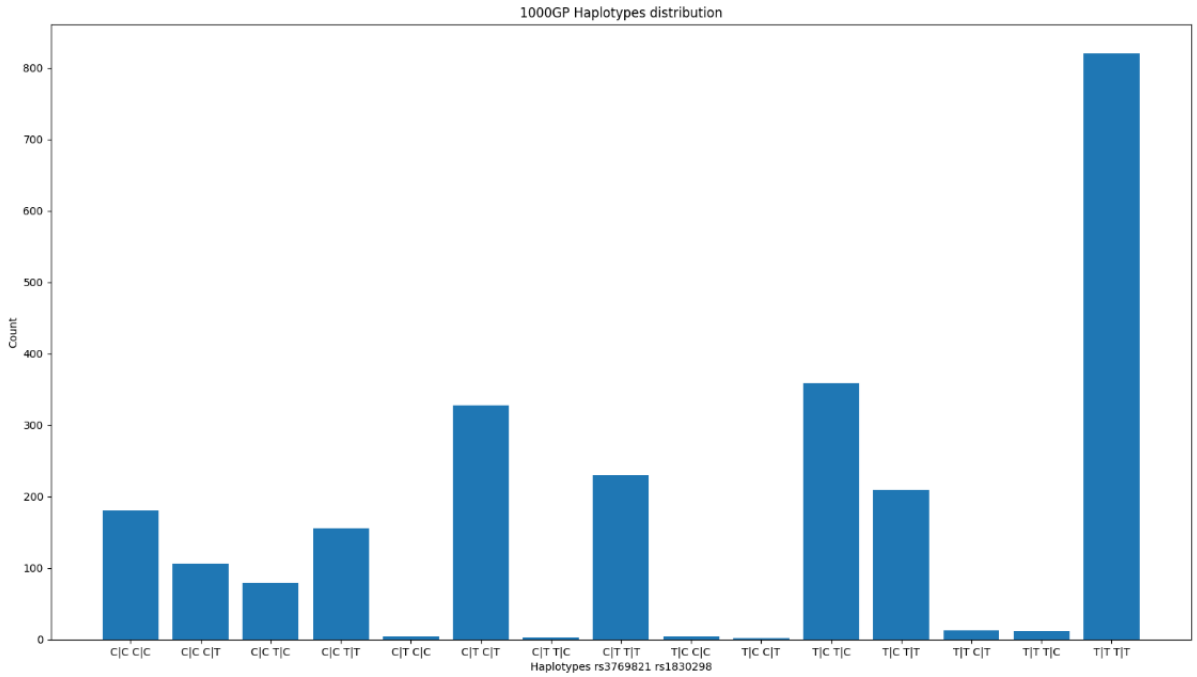

Figure S7. 1,000 Genomes Project genotypes for variants rs3769821 and rs1830298. Genotypes are phased. The first element in the pair refers to the two alleles of variant rs3769821 while the second element refers to the two alleles of variant rs1830298.

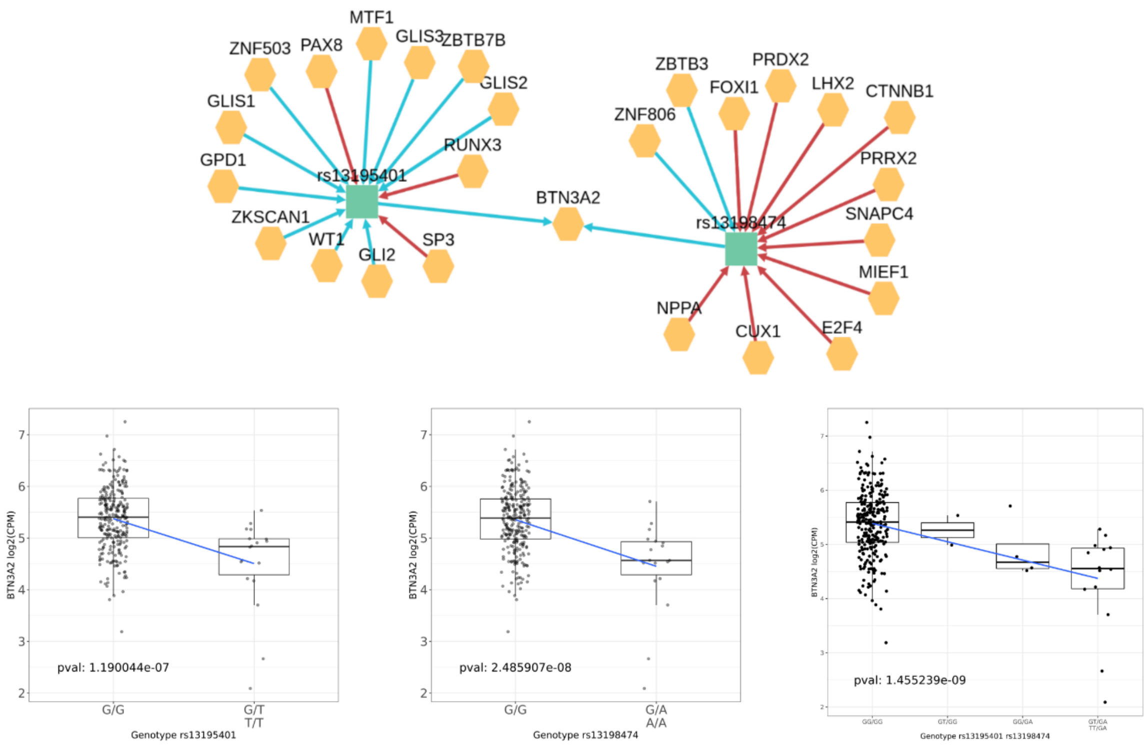

Figure S8. Variant-gene network (top) and effect of variants rs13195401 and rs13198474 on *BTN3A2* transcript in the breast tissue under the dominant model (bottom left and middle). Combined effect of the two variants (bottom, right) where the first boxplot contains samples with no dominant effect for both variants, the second has samples with a dominant effect for rs13195401 but no effect for rs13198474, the third has sample with a dominant effect for rs13198474 but no effect for rs13195401 and the fourth has samples with a dominant effect for both.

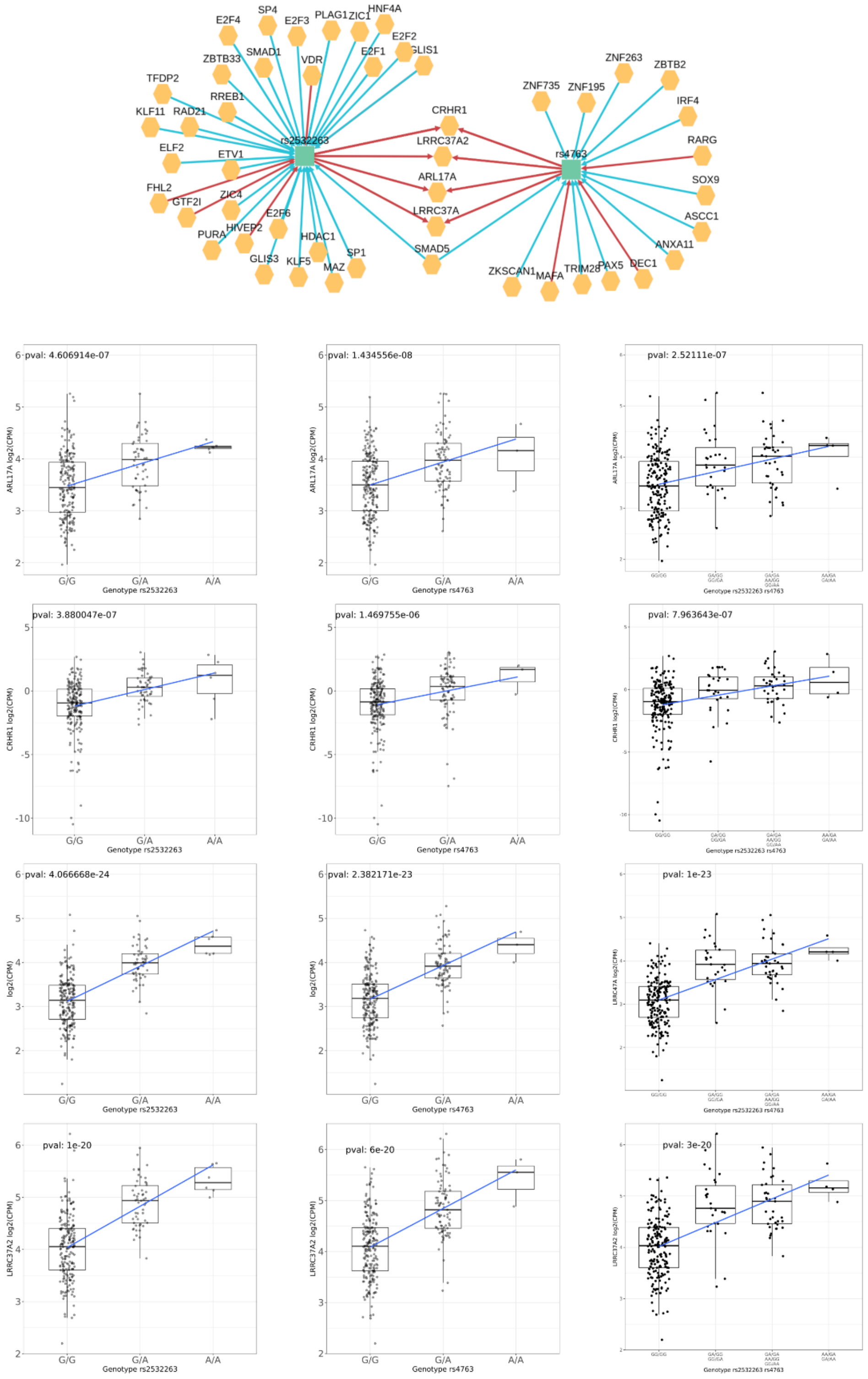

Figure S9. Variant-gene network of variants rs2532263 and rs4763 in breast tissue (top). Effects of rs2532263 and rs4763 on genes ARL17A, CRHR1, LRRC37A and LRRC37A2 under an additive model in breast tissue (bottom).

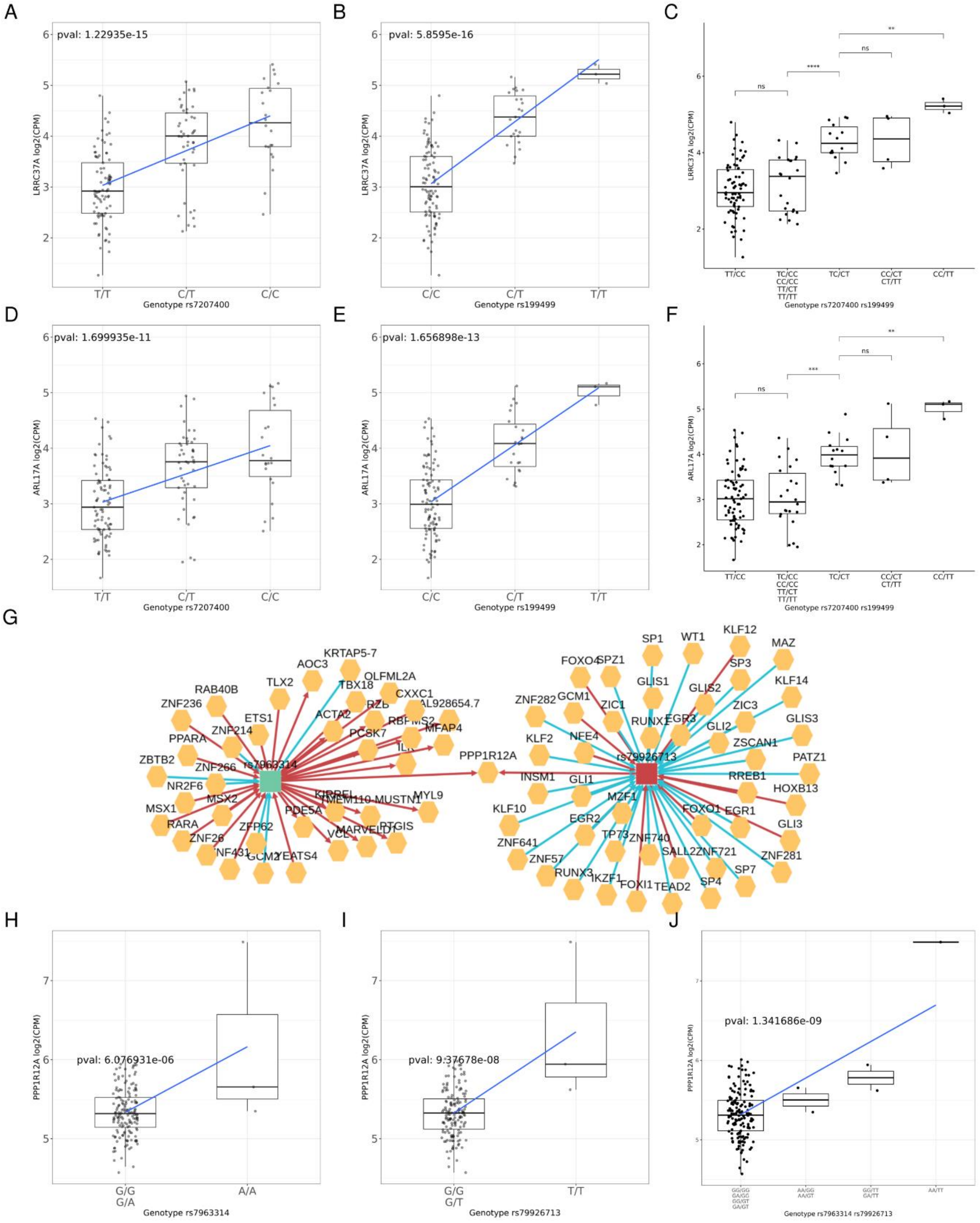

Figure S10. A) Effect of variant rs7207400 on the transcript level of *LRRC37A* under an additive model. **B)** Effect of variant rs199499 on the transcript level of *LRRC37A* under an additive model. **C)** Combined effect of the two variants rs7207400 (first pair of nucleotides in the label) and rs199499 (second pair) on *LRRC37A*. **D)** Effect of variant rs7207400 on the transcript level of *ARL17A* under an additive model. **E)** Effect of variant rs199499 on the transcript level of

ARL17A under an additive model. **F)** Combined effect of the two variants rs7207400 (first pair of nucleotides in the label) and rs199499 (second pair) on *ARL17A*. **G)** Variant-gene network of variants rs7963314 and rs79926713 in the brain tissue. **H)** Effect of variant rs7963314 on transcript *PPP1R12A* under a recessive model. **I)** Effect of variant rs79926713 on transcript *PPP1R12A* under a recessive model. **J)** Combined effect of the two variants where the first boxplot contains samples with no recessive effect for both variants, the second has samples with a recessive effect for rs7963314 but no effect for rs79926713, the third has sample with a recessive effect for rs79926713 but no effect for rs7963314 and the forth has samples with a recessive effect for both.

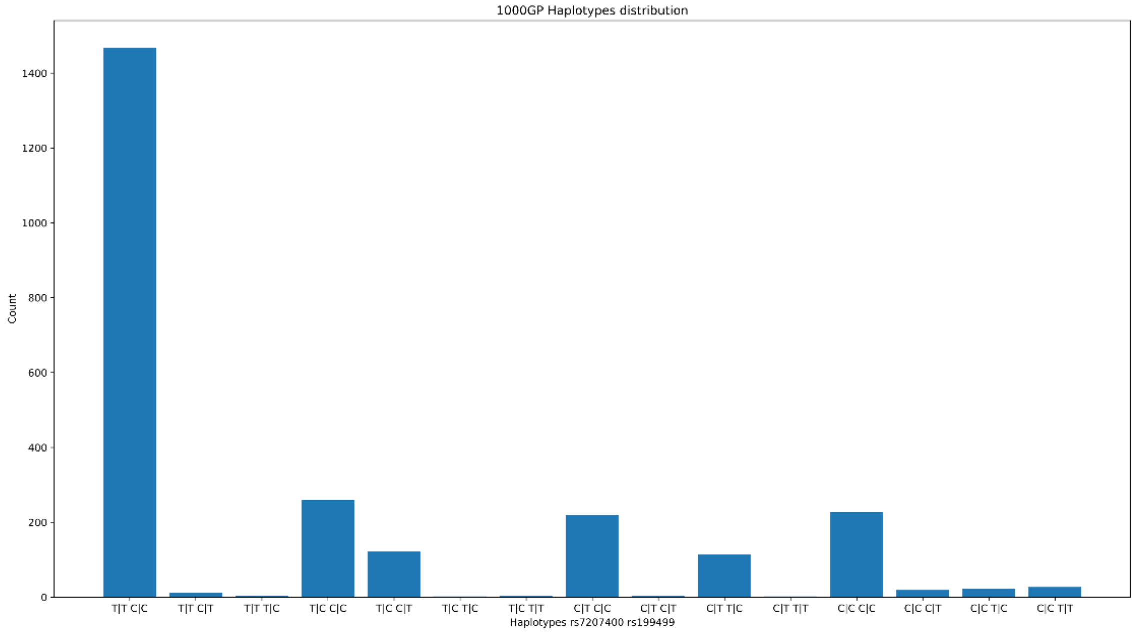

Figure S11. 1,000 Genomes Project genotypes for variants rs7207400 and rs199499. Genotypes are phased. The first element in the pair refers to the two alleles of variant rs7207400 while the second element in the pair refers to the two alleles of variant rs199499.
